# Expression and prognostic value of TRPM7 in canine mammary tumours

**DOI:** 10.1111/vco.12689

**Published:** 2021-05-04

**Authors:** Seulji Lee, Sungin Lee, Wan Hee Kim

**Affiliations:** ^1^ Department of Veterinary Clinical Sciences, College of Veterinary Medicine and Research Institute for Veterinary Science Seoul National University Seoul Republic of Korea; ^2^ Department of Veterinary Surgery Heamaru Referral Hospital Seongnam Republic of Korea

**Keywords:** dog, immunohistochemistry, ion channel, mammary gland tumour, transient receptor potential melastatin 7

## Abstract

Canine mammary gland tumour (CMTs) are one of the most commonly found tumours in intact female dogs. A previous study on canine mammary glands demonstrated the presence of the transient receptor potential melastatin 7 (TRPM7) ion channels in healthy canine mammary tissues. However, the significance of TRPM7 in CMT is not yet known. TRPM7 is a Ca^2+^ and Mg^2+^ permeable cation channel that contains a protein kinase domain. The aim of this study was to determine TRPM7 expression in 57 benign and malignant CMT tissues of dogs using immunohistochemistry (IHC) and evaluate its correlation with clinicopathological features and explore the potential prognostic value of TRPM7 in a prospective survival study. IHC analysis shows that TRPM7 was expressed in the cytoplasm of neoplastic epithelial cells. Moreover, TRPM7 expression was significantly associated with tumour malignancy (*P* = .027), Ki‐67 index (*P* < .0001) and metastasis (*P* < .0001). Survival curve analysis indicates that high TRPM7 expression was significantly associated with poor disease‐free (*P* = .035) and overall survival (*P* = .011) in malignant CMTs. Our results demonstrate that TRPM7 is expressed in CMTs and that its expression is positively correlated with clinicopathological parameters. Thus, TRPM7 was assumed to be a potential prognostic factor for CMTs.

## 1 INTRODUCTION

Canine mammary gland tumour (CMTs) account for 70% of all tumours in intact female dogs^1^, ^2^ and can be benign or malignant in nature. CMTs are hormone‐dependent and might recur after surgical removal or metastasize to other organs, in particular, the lymph node and lungs.^3^ The prognostic factors of cancer include histological type, tumour grade, mode of tumour growth, lymph node status and tumour size.^4^ These clinicopathological factors are important for evaluating and determining a prognosis as well as predicting the molecular cancer behaviour. In humans and animals, ion channels have been newly identified as prognostic factors that could potentially lead to the identification of new therapeutic targets. Deregulation of Ca^2+^ homeostasis has been implicated in mammary gland disease.^5^, ^6^, ^7^, ^8^ Moreover, Ca^2+^ and Mg^2+^ ion channels play an important role in cell proliferation, differentiation, apoptosis and oncogenesis.^9^, ^10^

Transient receptor potential (TRP) is a plasma membrane ion channel that regulates the permeability of Ca^2+^ and Mg^2+^ across the plasma membrane of animal cells.^7^ TRP channel activity is important for essential hallmarks of tumorigenesis. Therefore, TRP channels have not only been suggested as clinical markers but also as promising anticancer targets in recent years. TRP channels were first discovered in a TRP‐mutant strain of the fruit fly *Drosophila*, and are categorized into six subfamilies based on their amino acid sequences: the TRPC (canonical), TRPV (vanilloid), TRPM (melastatin), TRPA (ankyrin), TRPML (mucolipin) and TRPP (polycystin) channels.^11^ Dhennin‐Duthille et al^10^ reported that increased expression of TRP channels is a useful biomarker for the diagnosis, prognosis and/or treatment of human breast ductal adenocarcinoma. Accumulating evidence has indicated that increased expression of TRP channels can be used as a biomarker for several human malignancies.^12^, ^13^, ^14^ TRPC, TRPM and TRPV expression levels were shown to be correlated with malignant growth and cancer progression.^15^

Transient receptor potential melastatin 7 (TRPM7) channel is widely expressed in various organs, including the heart, lung, liver, brain and spleen.^16^ It is overexpressed in various types of cancers, such as ovarian carcinoma, retinoblastoma, neck and head carcinoma, prostate cancer, lung cancer and pancreatic adenocarcinoma.^17^, ^18^, ^19^, ^20^ Furthermore, an increased expression of TRPM7 channel is correlated with breast cancer progression and metastasis.^21^ Another study showed that siRNA‐mediated knockdown of TRPM7 expression in MCF‐7 cells impairs biological functions, highlighting the importance of TRPM7 expression in human breast cancer epithelial cells.^9^

Aberrant TRPM7 expression in human breast and pancreatic cancers is closely correlated with clinicopathological parameters, such as tumour grade, Ki‐67 proliferation index and patient survival time.^22^ A previous study had proven that TRPM7 is necessary for pancreatic cancer cell invasion.^23^ Despite abundant knowledge on TRPM7‐related carcinogenic pathways in human breast cancer, its role in CMT pathogenesis remains poorly understood. In addition, a previous study described the presence and distribution of TRPM7 channels in canine normal mammary gland tissue using RT‐PCR, immunohistochemistry (IHC) and western blotting.^24^ Since TRPM7 has not been studied in the context of clinical value, its prognostic value in CMTs is still unknown.

In this study, we aimed to investigate the presence of TRPM7 in CMTs by IHC and to evaluate the correlation between TRPM7 and clinicopathological features of dogs to explore the potential prognostic value of TRPM7 in survival study.

## 2 MATERIALS AND METHODS

### 2.1 Tissue samples

A total of 57 tissue samples were collected from dogs diagnosed with CMTs at Seoul National University Veterinary Medical Teaching Hospital between January 2010 and December 2017, based on the examination of H&E stained sections by the Veterinary Pathology Laboratory of Seoul National University. Of these, 21 and 36 were from malignant and benign tumours, respectively. Informed consent was obtained from the owners for the collection and use of tissue samples. The protocol was reviewed and approved by the Institutional Animal Care and Use Committee of Seoul National University. The data used in this study included only those for animals with mammary gland tumours (primary lesion) after being diagnosed with fine needle aspiration (FNA) or biopsy prior to surgical removal. All CMT tissues were fixed in 10% neutral buffered formalin for 48 hour at room temperature and embedded in paraffin blocks. Thereafter, 4‐μm sections were cut and slides were stained with haematoxylin‐eosin for diagnostic purposes. Each slide was evaluated under a microscope and classified according to the diagnostic criteria of Goldschmidt et al^25^ for classification and of Peña et al^26^ for the grading system. Further, Ki‐67 proliferative index (≤15%, >15% of the cells) and tumour size (≤3 cm, >3 cm) were also evaluated. Animals with a single mammary gland tumour as well as those with multiple tumours were included in this study. When dogs presented with more than one malignant neoplasm, the tumour with the more aggressive clinical and pathological features was selected, as recommended by Sorenmo et al.^1^ In addition, animals with mammary gland tumours and other malignancies detected by various screening tests prior to surgical removal or those already undergoing chemotherapy were excluded.

### 2.2 Immunohistochemistry

The paraffin‐embedded tissues were sectioned at 4‐μm intervals using a microtome (Leica Microtome HM355S, Plymouth, Minnesota), deparaffinized in xylene twice for 5 minutes each, and rehydrated in graded alcohol (100% twice, 95%, 90%, 80% and 70% once for 3 minutes each). Antigen retrieval was carried out using a 2100‐retriever pressure cooker (PickCell Laboratories, Amsterdam, Netherlands) in a 10 mM citrate acid (pH 6) buffer for 20 minutes. Endogenous peroxidase activity was quenched by incubation with 3% H_2_O_2_ for 30 minutes. The sections were treated with normal horse serum (S‐2012, Vector, CA, USA) for 20 minutes to block non‐specific binding and incubated overnight at 4°C with goat anti‐TRPM7 antibody (1:300; ab729, Abcam, Cambridge, Massachusetts) and rabbit anti‐Ki‐67 polyclonal antibody (1:500; PA5‐19462, Invitrogen Ltd, Paisley, England). The antibody used against TRPM7 was previously validated for canine tissues.^24^ The sections were subsequently incubated with secondary antibodies HRP anti‐goat IgG (ImmPACTTM, Vector, California) and HRP anti‐rabbit IgG (ImmPACTTM, Vector) for 1 hour. The slides were incubated in 3,3′‐diaminobenzidine tetrahydrochloride (ImmPACTTM) diaminobenzidine (DAB) peroxidase substrate kit, Vector) for 90 seconds and the reaction was stopped by immersion in distilled water. In a previous study, normal C57BL/6J mouse brain tissues were used as positive control.^24^, ^27^ Negative control samples were incubated in the absence of the primary antibodies to rule out non‐specific binding by the secondary antibodies. The tissue sections were counterstained with Mayer's haematoxylin, dehydrated in graded alcohol and cleared in xylene. The slides were washed with PBS between each procedure. Immunostained slides were scanned using an Olympus BX51 microscope (Olympus, Japan) with appropriate light filters (Tucsen, Fuzhou, China).

### 2.3 Quantitation of IHC staining

IHC results were analysed using Aperio ImageScope version 12.3.0.5056 (Aperio, Vista, California). IHC slide images were analysed using the Aperio program. These algorithms used colour de‐convolution to separate DAB from the haematoxylin counterstain. The algorithm to determine the intensity of cytoplasmic and nuclear staining for each slide was used to calculate the staining intensity and percent of target labelled by digitally analysing the colour intensity. The output staining intensities ranged from 0 (negative) to +3 (strong positive) and were correlated with conventional manual scoring methods.^28^

Aperio Cytoplasm V2 algorithm was used to analyse cytoplasmic positivity of TRPM7 expression, based on criteria for TRPM7 in human breast cancer.^29^, ^30^ The positive expression of TRPM7 in cytoplasm was quantitatively assessed, for each sample in 10 randomly selected high‐power fields (400×), as follows: 0 (negative), +1 (weakly positive), +2 (moderately positive) and + 3 (strongly positive). Staining intensities for TRPM7 expression ranged from 0 to 3 and were correlated with conventional manual scoring methods. Scores of +2 and + 3 represented TRPM7 overexpression, and the percentage of cases overexpressing TRPM7 was calculated. We categorized TRPM7 expression as low (0, +1) or high (+2, +3) for analysis.

Aperio Nuclear V9 algorithm was used to analyse nuclear positivity of Ki‐67. An algorithm for analysing nuclear immunoreactivity was used to measure the percentage of immunoreactive cells.^31^, ^32^ All positively stained cells in the whole cell area were counted and the fraction of positive cells was calculated as the number of positive cells/1000. High Ki‐67 index value was defined as ≥15% independent of nuclear staining intensity. A ≥ 15% cut‐off threshold was used in accordance with Kadthur et al^33^ in which a comprehensive standardized statistical analysis was used to form low and high‐risk groups based on appropriate cut‐off value to indicate the prognosis of CMTs.

### 2.4 Follow‐up data

All dogs with CMTs were evaluated before surgery, 3 weeks after surgery, and every 3 months for at least 2 years. Owners were instructed to contact the hospital at any time, even if it was not related with CMT, whenever they discovered abnormalities. Assessment of metastasis and recurrence of tumours was carried out by physical examination, thoracic radiograph (three views), ultrasound (abdomen), FNA, biopsy, autopsy and/or CT scan (if required) at the Seoul National University hospital and/or referred to another animal hospital.

Whenever new mammary gland lesions were discovered, lymph nodes were considered clinically abnormal or lesions were detected in any other organ, additional examinations (eg, FNA, excisional biopsy and CT scan) were performed to rule out other neoplasms and/or to identify local recurrences or metastatic disease.

### 2.5 Statistical analysis

Correlation between TRPM7 and clinicopathological parameters was analysed using Fisher exact test, the chi‐square and the linear‐by‐linear association test. Kaplan‐Meier survival curves were plotted and compared using the log‐rank test. All the statistical analyses were performed using SPSS software (SPSS, Chicago, Illinois).

Disease‐free survival (DFS) was defined as the interval (months) from primary surgical treatment to the date of detection of the first local recurrence or development of metastases. Overall survival (OS) was calculated from the date of primary surgical treatment to the time of death from the cancer. In the OS study, the dogs were censored if and when they died from causes unrelated to mammary tumours, were lost to follow‐up, or were alive 2 years after surgery. In the DFS study, the dogs were censored if and when they were lost to follow‐up, died from causes unrelated to mammary tumours before developing signs of metastatic disease, or were free of metastases 24 months post‐surgery. Each tick mark represents the time at which a patient was censored. A *P* < .05 was considered to be statistically significant.

## 3 RESULTS

### 3.1 Dogs

A total of 57 dogs diagnosed with CMTs were included in this study. The signalment data are presented in Table 1. The median age of dogs with benign CMTs was 11.00 years (range: 6‐16 years) and was similar to that of dogs with malignant CMTs (11.94 years; range: 6‐15 years). Forty dogs were sexually intact females, whereas 17 dogs were spayed females. The major breeds were Yorkshire Terrier (n = 17) and Maltese (n = 10). Benign CMT samples were classified into the following three groups: complex adenomas (n = 19), simple adenomas (n = 8) and benign mixed tumours (n = 9). Malignant CMT samples were classified into the following three groups: simple carcinomas (n = 11), complex carcinomas (n = 8) and mixed carcinoma (n = 2) (Table 1). Metastases were confirmed in 11 samples of malignant CMTs.

**TABLE 1:** Comparison of signalment data (age, sex, breed and histologic diagnosis) of benign and malignant mammary gland tumours in 57 dogs

	Benign tumours (n = 36)	Malignant tumours (n = 21)
Median age (range)	11.00 (6‐16)	11.94 (6‐15)
Sex (n)	Intact female (26) Spayed female (10)	Intact female (14) Spayed female (7)
Breed (n)	Yorkshire Terrier (14) Maltese (7) Poodle (3) Cocker Spaniel (4) Mixed (2) Schnauzers (2) Chihuahua (1) Miniature Pinscher (1) Boston Terrier (1) Shih‐tzu (1)	Maltese (7) Yorkshire Terrier (3) Poodle (3) Shih‐tzu (4) Jindo (1) Malinois (1) Cocker Spaniel (1) Dachshund (1)
Histologic type (n)	Complex adenoma (19) Simple adenoma (8) Benign mixed tumour (9)	Simple carcinomas (11) Complex carcinomas (8) Mixed carcinomas (2)

### 3.2 Immunolocalization

**FIGURE 1:**
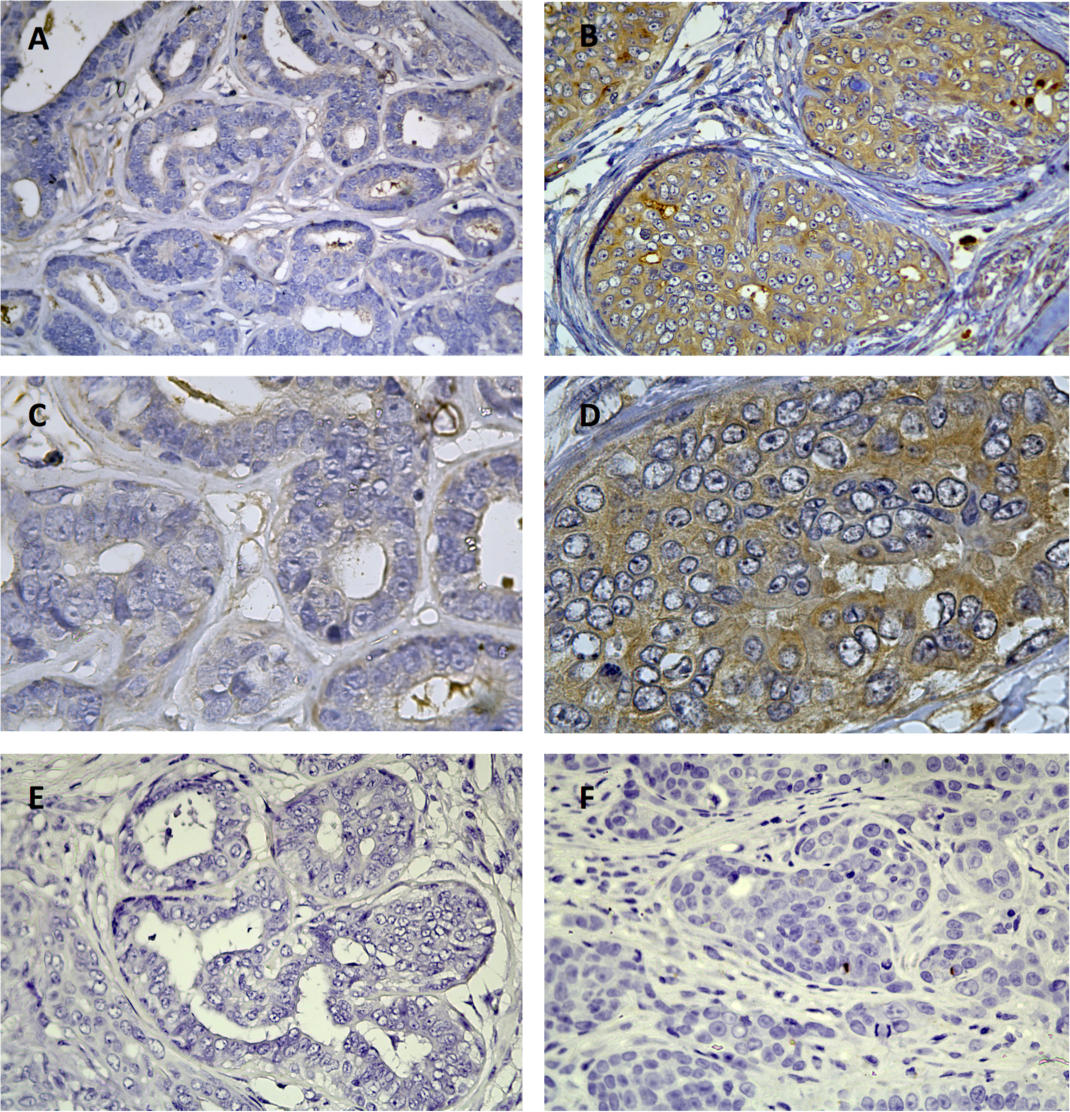
Immunohistochemical staining of TRPM7 in canine mammary gland tumour (CMT) tissues. A and C, Benign CMT (simple adenoma) with low‐TRPM7 expression (weak positive). B and D, Malignant CMT (Grade III carcinoma) with high‐TRPM7 expression (strong positive). E, No specific staining was observed in the negative control samples of benign CMTs. F, No specific staining was observed in the negative control of malignant CMTs. Sections were counterstained with haematoxylin. (A, B, E, F original magnification ×400; C and D original magnification ×1000). CMT, canine mammary gland tumour; TRPM7, transient receptor potential melastatin 7

IHC staining showed TRPM7 expression in the cytoplasm of neoplastic epithelial cells. Cytoplasmic immunoreactivity in benign tumours was weak compared to that in malignant tumours. In addition, no immunoreactivity was observed in myoepithelial cells of complex adenomas and mesenchymal areas of benign mixed tumours. A cell population was observed in the mesenchymal area, and TRPM7 expression in these cells was higher than that in the adjacent non‐cancerous cells (Figure 1A‐D).

### 3.3 Relationship between TRPM7 expression and tumour grade or pathological factor

Of the malignant MGTs, 66.67% showed high‐TRPM7 expression. Furthermore, 57% of grade II MGTs and 87.5% of grade III MGTs showed high‐receptor expression. In malignant tumours, 100% of mixed carcinomas, 62.5% of complex carcinomas and 54% of simple carcinomas showed high‐receptor expression. In addition, 12.5% of simple adenomas, 5% of complex adenomas and 11.1% of benign mixed tumours showed high‐receptor expression. Moreover, there was significant correlation between TRPM7 receptor expression and tumour grade (*P* = .027), metastasis (*P* < .0001) and Ki‐67 index (*P* < .0001), but not with other clinicopathologic parameters such as tumour size and histological diagnosis (Table 2).

**TABLE 2:** Association between TRPM7 expression and clinicopathological parameters

Variable	TRPM7 expression			
	Number of tumours	Low	High	*P*
Tumour size				
≤3 cm	35	32	3	
>3 cm	22	8	14	.504
Benign CMT				
Simple adenoma	8	7	1	
Complex adenoma	19	18	1	
Mixed tumour	9	8	1	.942
Malignant CMT				
Simple carcinoma	11	5	6	
Complex carcinoma	8	3	5	
Mixed carcinoma	2	0	2	.296
Histological grade				
I	6	3	3	
II	7	3	4	
III	8	1	7	.027^a^
Metastases				
Absent	10	10	0	
Present	11	0	11	<.0001^a^
Ki‐67 labelling index				
≤15%	37	35	2	
>15%	20	5	15	<.0001^a^

Abbreviations: CMT, canine mammary gland tumour; TRPM7, transient receptor potential melastatin 7.

^a^ A P‐value < .05 indicates that at high expression of TRPM7, the clinicopathological variables considered to be a statistically significant.

### 3.4 Correlation between TRPM7 overexpression and clinical outcome

**FIGURE 2:**
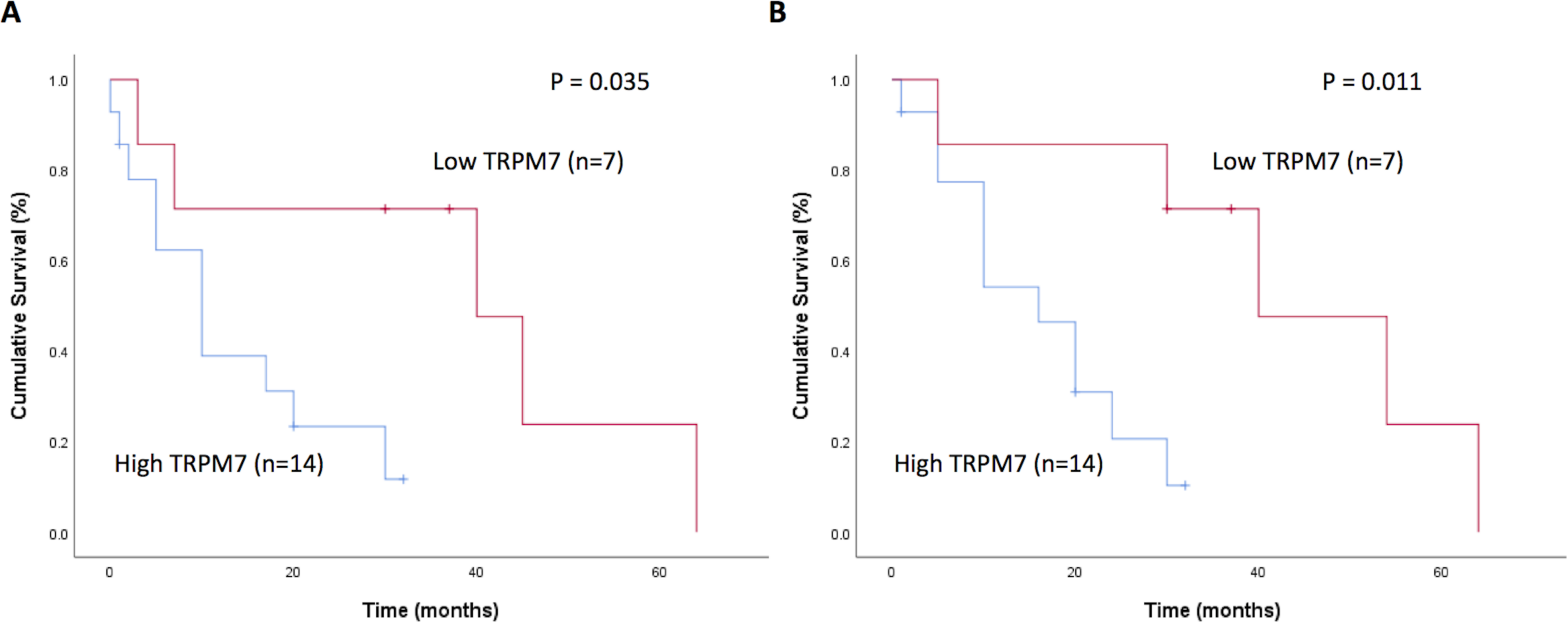
Kaplan‐Meier survival curves of 21 dogs with malignant CMTs based on TRPM7 expression status for A, disease‐free survival (median: 18 months) and B, overall survival (median 22 months). TRPM7, transient receptor potential melastatin 7

The prognostic value of TRPM7 overexpression in malignant CMTs was determined by Kaplan‐Meier analysis. In malignant CMTs, 15 dogs died (13 as a result of CMTs) and six dogs were censored. All of the 21 malignant CMT samples were classified into two TRPM7 groups: high (n = 14) and low (n = 7). Survival curves showed a significant difference between the high and low‐TRPM7 expression groups; the high‐TRPM7 expression group was associated with poor DFS and shorter OS. The median DFS and OS were 18 and 22 months, respectively (Figure 2A,B). Dogs with high TRPM7 expression levels had worse prognosis than those with low expression levels of TRPM7 in CMTs.

## 4 DISCUSSION

TRPM7 is a ubiquitously expressed protein and plays a prominent role in early embryogenesis and organogenesis.^34^, ^35^ It is involved in cell cycle progression, adhesion, survival and migration of cancer cells.^36^ Although several studies have demonstrated the expression of TPRM7 in MCF‐7 cells or tumour tissues through various experiments, to our knowledge, this is the first study to evaluate TRPM7 expression in CMTs. In contrast to immunoreactivity at the apical membrane of ductal epithelial cells of canine normal mammary gland, TRPM7 was diffusely expressed in the cytoplasm at elevated levels in higher grade CMTs. This pattern was also observed in the pancreas wherein TRPM7 was expressed in apical plasma membrane of pancreatic ductal epithelia and in the cytoplasm of pancreatic adenocarcinoma cells.^37^ We investigated whether other TRP channels show differential immunoreactivity (plasma membrane or cytoplasm) in normal cell and tumour state. TRPM8 protein was localized to the plasma membrane of cells in the normal prostate tissue, whereas its channel showed severely internalized pattern of TRPM8 in tumour tissues.^38^, ^39^ Taken together, these data suggest that IHC staining results of pancreatic and prostate in human normal and cancer cells differ from that of human mammary gland results; however, unlike in human mammary gland tissues, TRPM7 channels are internalized in CMTs and are expressed in the apical membrane of ductal epithelial cells of normal mammary gland tissue. Previous studies have demonstrated that TRPM7 mRNA and protein are expressed in canine normal mammary glands.^24^ Despite the vast number of studies on TRPM7 over the past decade, its function and mechanism of action are not fully understood. TRPM7 expression can vary according to the tissue type and its localization and gating in plasma or intracellular membranes.^40^

The correlation between TRPM7 expression and clinicopathological parameters (tumour size, histological diagnosis, tumour grade, metastasis and Ki‐67) was assessed. These parameters are widely known as prognostic factors in CMTs.^41^ Our results showed a statistically significant association between TRPM7 overexpression and higher tumour grade, metastasis and higher Ki‐67. The positive correlation between tumour progression and TRPM7 expression may result from the role of TRPM7 in cancer development. As mentioned previously, TRPM7 plays different roles during cancer progression. TRPM7 is required for cell proliferation and migration as well as epithelial‐mesenchymal transition in the early stages and for the regulation of Ca^2+^ and Mg^2+^ homeostasis during cell proliferation, migration and invasion in the advanced‐stage. In addition, aggressive tumours require TRPM7 channel activity and interaction with cytoskeletal proteins.^42^

Our findings demonstrated that TRPM7 overexpression is correlated with poor DFS and OS. These results not only suggest a prominent role of TRPM7 in cell cycle regulation and proliferation in CMTs but also imply an application of TRPM7 as a valuable prognostic marker. Additionally, human studies have shown that TRPM7 overexpression in patients with breast, ovarian and pancreatic cancer is significantly associated with OS and DFS.^21^, ^43^, ^44^

It should be noted that this study has several limitations. First, the analysed sample size of benign and malignant CMT is relatively small. A larger sample size would help to obtain reliable results while allowing multi‐perspective analysis. Especially, in benign tumours, the correlation between histopathological diagnosis and pathological features was limited as it was difficult to obtain statistical data owing to the small number of benign tumours showing overexpression of TRPM7. Second, this study did not evaluate the relative mRNA and protein levels by real‐time PCR and western blotting. Further investigations are required to address these issues to advance our understanding of TRPM7 function and regulation in health and disease.

Previous results and the current study have provided basic data for future studies to evaluate TRPM7 expression and OS in canine mammary gland tumour. These findings suggest that TRPM7 might have prognostic value and, ultimately, we need to discuss the potential function of TRPM7 channel‐kinase as a biomarker and therapeutic target for veterinary oncology.^45^

## 5 CONCLUSION

In this study, we showed that TRPM7 is expressed in the cytoplasm of benign and malignant CMTs. We observed higher TRPM7 expression in malignant CMTs than in benign CMTs, indicating that TRPM7 is involved in cancer progression. Furthermore, we demonstrated that TRPM7 overexpression is positively associated with prognostic factors such as tumour grade, Ki‐67 index and metastasis. In addition, our findings demonstrate that high‐TRPM7 expression is significantly associated with DFS and OS. However, further studies are required to understand the mechanism by which TRPM7 overexpression promotes the development of CMTs.

### ACNOWLEDGEMENTS

This work was carried out with the support of “Cooperative Research Program of Center for Companion Animal Research (Project No. PJ01499001)” Rural Development Administration, Republic of Korea. The Research Institute for Veterinary Science, Seoul National University supported the publication charges for this research.

## CONFLICT OF INTEREST

The authors have declared that no competing interests exist. The funders had no role in the design of the research; in the collection, analyses or interpretation of data; in the writing of the article, or in the decision to publish the results.

## ETHICS APPROVAL

This study is a retrospective investigation carried out on archived tissue samples of CMT. The protocol was reviewed and approved by the Institutional Animal Care and Used Committee of Seoul National University (SNU‐190314‐6‐1). Informed consent was obtained from the owners for the collection and use of tissue samples.

## Supporting information

**Supplementary Table 1** The average cytoplasmic intensity scores for the selected region were calculated based on the thresholds

## Data Availability

The data that support the findings of this research are available from the corresponding author upon reasonable request.

## References

[1] Sorenmo KU, Worley DR, Goldschmidt MH. Tumors of the mammary gland. In: Withrow SJ, Vail DM, Rodney LP, eds. Small Animal Clinical Oncology. 5th ed. St. Louis, MO: Elsevier Saunders; 2013:538‐556.

[2] Johnston SA, Tobias KM. Veterinary Surgery: Small Animal Expert Consult. 2nd ed. St. Louis, MO: Elsevier; 2018.

[3] Nassiri MR, Hosseini SA, Ghovvati S, Zabetian M. Evaluation of estrogen receptor α and ß genes expression in normal and neoplastic mammary gland in dogs by real‐time PCR. Curr Pharmacogen Person Med. 2018;16:140‐146.

[4] Santos AA, Lopes CC, Ribeiro JR, et al. Identification of prognostic factors in canine mammary malignant tumours: a multivariable survival study. BMC Vet Res. 2013;9:1. 23289974 10.1186/1746-6148-9-1 PMC3542312

[5] Lee WJ, Monteith GR, Roberts‐Thomson SJ. Calcium transport and signaling in the mammary gland: targets for breast cancer. Biochim Biophys Acta. 2006;1765:235‐255. 16410040 10.1016/j.bbcan.2005.12.001

[6] VanHouten J. Calcium sensing by the mammary gland. J Mammary Gland Biol Neoplasia. 2005;10:129‐139. 16025220 10.1007/s10911-005-5396-y

[7] Sergeev I. Calcium signaling in cancer and vitamin D. J Steroid Biochem Mol Biol. 2005;97:145‐151. 16081284 10.1016/j.jsbmb.2005.06.007

[8] Lee WJ, Roberts‐Thomson SJ, Holman NA, May FJ, Lehrbach GM, Monteith GR. Expression of plasma membrane calcium pump isoform mRNAs in breast cancer cell lines. Cell Signal. 2002;14:1015‐1022. 12359307 10.1016/s0898-6568(02)00049-9

[9] Guilbert A, Gautier M, Dhennin‐Duthille I, Haren N, Sevestre H, Ouadid‐Ahidouch H. Evidence that TRPM7 is required for breast cancer cell proliferation. Am J Physiol Cell Physiol. 2009;297:C493‐C502. 19515901 10.1152/ajpcell.00624.2008

[10] Dhennin‐Duthille I, Gautier M, Faouzi M, et al. High expression of transient receptor potential channels in human breast cancer epithelial cells and tissues: correlation with pathological parameters. Cell Physiol Biochem. 2011;28:813‐822. 22178934 10.1159/000335795

[11] Park HS, Hong C, Kim BJ, So I. The pathophysiologic roles of TRPM 7 channel. Korean J Physiol Pharmacol. 2014;18:15‐23. 24634592 10.4196/kjpp.2014.18.1.15 PMC3951819

[12] Chen JP, Luan Y, Yu R, Zhang Z, Zhang J, Wang W. Transient receptor potential (TRP) channels, promising potential diagnostic and therapeutic tools for cancer. Biosci Trends. 2014;8:1‐10. 24647107 10.5582/bst.8.1

[13] Bödding M. TRP proteins and cancer. Cell Signal. 2007;19:617‐624. 17029734 10.1016/j.cellsig.2006.08.012

[14] Gkika D, Prevarskaya N. Molecular mechanisms of TRP regulation in tumor growth and metastasis. Mol Cell Res. 2009;1793:953‐958. 10.1016/j.bbamcr.2008.11.010 19103233

[15] Ouadid‐Ahidouch H, Dhennin‐Duthille I, Gautier M, Sevestre H, Ahidouch A. TRP channels: diagnostic markers and therapeutic targets for breast cancer? Trends Mol Med. 2013;19:117‐124. 23253476 10.1016/j.molmed.2012.11.004

[16] Nadler MJS, Hermosura MC, Inabe K, et al. LTRPC7 is a mg•ATP‐regulated divalent cation channel required for cell viability. Nature. 2001;411:590‐595. 11385574 10.1038/35079092

[17] Wang J, Liao Q‐J, Zhang Y, et al. TRPM7 is required for ovarian cancer cell growth, migration and invasion. Biochem Biophys Res Commun. 2014;454:547‐553. 25450691 10.1016/j.bbrc.2014.10.118

[18] Jiang J, Li M‐H, Inoue K, Chu X‐P, Seeds J, Xiong Z‐G. Transient receptor potential melastatin 7‐like current in human head and neck carcinoma cells: role in cell proliferation. Cancer Res. 2007;67:10929‐10938. 18006838 10.1158/0008-5472.CAN-07-1121 PMC2398732

[19] Sun Y, Selvaraj S, Varma A, Derry S, Sahmoun AE, Singh BB. Increase in serum Ca2+/Mg2+ ratio promotes proliferation of prostate cancer cells by activating TRPM7 channels. J Biol Chem. 2013;288:255‐263. 23168410 10.1074/jbc.M112.393918 PMC3537020

[20] Gautier M, Perrière M, Monet M, et al. Recent advances in oncogenic roles of the TRPM7 chanzyme. Curr Med Chem. 2016;23:4092‐4107. 27604090 10.2174/0929867323666160907162002

[21] Middelbeek J, Kuipers AJ, Henneman L, et al. TRPM7 is required for breast tumor cell metastasis. Cancer Res. 2012;72:4250‐4061. 22871386 10.1158/0008-5472.CAN-11-3863

[22] Zhou W, Guo S, Xiong Z, Liu M. Oncogenic role and therapeutic target of transient receptor potential melastatin 7 channel in malignancy. Expert Opin Ther Targets. 2014;18:1177‐1196. 25069584 10.1517/14728222.2014.940894

[23] Yee NS, Kazi AA, Li Q, Yang Z, Berg A, Yee RK. Aberrant over‐expression of TRPM7 ion channels in pancreatic cancer: required for cancer cell invasion and implicated in tumor growth and metastasis. Biol Open. 2015;4:507‐514. 25770184 10.1242/bio.20137088 PMC4400593

[24] Sungin L, Seulji L, Aeri L, et al. The presence and distribution of TRPM7 in the canine mammary glands. Animals. 2020;10:466. 10.3390/ani10030466 PMC7142925 32168794

[25] Goldschmidt M, Peña L, Rasotto R, Zappulli V. Classification and grading of canine mammary tumors. Vet Pathol. 2011;48:117‐131. 21266722 10.1177/0300985810393258

[26] Peña L, Andrés PD, Clemente M, Cuesta P, Perez‐Alenza M. Prognostic value of histological grading in noninflammatory canine mammary carcinomas in a prospective study with two‐year follow‐up: relationship with clinical and histological characteristics. Vet Pathol. 2013;50:94‐105. 22688585 10.1177/0300985812447830

[27] Etem EO, Bal R, Akağaç AE, et al. The effects of hydrated C (60) fullerene on gene expression profile of TRPM2 and TRPM7 in hyperhomocysteinemic mice. J Recep Signal Trans. 2014;34(4):317‐324. 10.3109/10799893.2014.896381 24646197

[28] Singh R, Gupta P, Kloecker GH, Singh S, Lillard JW Jr. Expression and clinical significance of CXCR5/CXCL13 in human non‐small cell lung carcinoma. Int J Oncol. 2014;45:2232‐2240. 25271023 10.3892/ijo.2014.2688 PMC4215579

[29] Piga I, Verza M, Montenegro F, et al. In situ metabolic profiling of ovarian cancer tumor xenografts: a digital pathology approach. Front Oncol. 2020;10:1277. 32974128 10.3389/fonc.2020.01277 PMC7466758

[30] Lucien F, Lac V, Billadeau DD, Borgida A, Gallinger S, Leong HS. Glypican‐1 and glycoprotein 2 bearing extracellular vesicles do not discern pancreatic cancer from benign pancreatic diseases. Oncotarget. 2019;10(10):1045‐1055. 30800217 10.18632/oncotarget.26620 PMC6383691

[31] Dowsett M, Nielsen TO, A'Hern R, et al. Assessment of Ki67 in breast cancer: recommendations from the international Ki67 in breast cancer working group. J Natl Cancer Inst. 2011;103:1656‐1664. 21960707 10.1093/jnci/djr393 PMC3216967

[32] Gasperetti F, Ferro A, Cuorvo LV, et al. Proliferative activity in human breast cancer: Ki‐67 automated evaluation and the influence of different Ki‐67 equivalent antibodies. Diagn Pathol. 2011;6:S7. 21489202 10.1186/1746-1596-6-S1-S7 PMC3073225

[33] Kadthur JC, Rao S, Laxmikanth SM, Sonnahallipura BM, Thimmanahalli DS. Prognostic value of Ki 67 proliferation antigen in canine malignant mammary gland tumours. Braz J Vet Pathol. 2011;4:36‐40.

[34] Jie J, Long‐Jun W, Janice J, et al. The channel kinase, TRPM7, is required for early embryonic development. Proc Natl Acad Sci. 2012;109:E225. 22203997 10.1073/pnas.1120033109 PMC3277139

[35] Duan J, Li Z, Li J, et al. Structure of the mammalian TRPM7, a magnesium channel required during embryonic development. Proc Natl Acad Sci. 2018;115:E8201‐E8210. 30108148 10.1073/pnas.1810719115 PMC6126765

[36] Yee NS, Chan AS, Yee JD, Yee RK. TRPM7 and TRPM8 ion channels in pancreatic adenocarcinoma: potential roles as cancer biomarkers and targets. Scientifica. 2012;2012:415158. 24278689 10.6064/2012/415158 PMC3820452

[37] Yee NS, Zhou W, Liang IC. Transient receptor potential ion channel Trpm7 regulates exocrine pancreatic epithelial proliferation by Mg2+ −sensitive Socs3a signaling in development and cancer. Dis Model Mech. 2011;4:240‐254. 21183474 10.1242/dmm.004564 PMC3046099

[38] Bidaux G, Flourakis M, Thebault S, Zholos A. Prostate cell differentiation status determines transient receptor potential melastatin member 8 channel subcellular localization and function. J Clin Invest. 2007;117:1647‐1657. 17510704 10.1172/JCI30168 PMC1866249

[39] Asuthkar S, Demirkhanyan L, Mueting S, Cohen A, Zakharian E. High‐throughput proteome analysis reveals targeted TRPM8 degradation in prostate cancer. Oncotarget. 2017;8:12877‐12890. 28039451 10.18632/oncotarget.14178 PMC5355063

[40] Krapivinsky G, Krapivinsky L, Manasian Y, Clapham DE. The TRPM7 chanzyme is cleaved to release a chromatin‐modifying kinase. Cell. 2014;157:1061‐1072. 24855944 10.1016/j.cell.2014.03.046 PMC4156102

[41] Misdorp W, Hart AAM. Prognostic factors in canine mammary cancer. J Natl Cancer Inst. 1976;56:779‐786. 1255797 10.1093/jnci/56.4.779

[42] Isabelle Dhennin‐Duthille MG, Korichneva I, Ouadid‐Ahidouch H. TRPM7 involvement in cancer: a potential prognostic factor. Magnes Res. 2014;27:103‐112. 25367030 10.1684/mrh.2014.0367

[43] Wang J, Xiao L, Luo C‐H, et al. Overexpression of TRPM7 is associated with poor prognosis in human ovarian carcinoma. Asian Pac J Cancer Prev. 2014;15:3955‐3958. 24935580 10.7314/apjcp.2014.15.9.3955

[44] Rybarczyk P, Gautier M, Hague F, et al. Transient receptor potential melastatin‐related 7 channel is overexpressed in human pancreatic ductal adenocarcinomas and regulates human pancreatic cancer cell migration. Int J Cancer. 2012;131:E851‐E861. 22323115 10.1002/ijc.27487

[45] Yee NS. Role of TRPM7 in cancer: potential as molecular biomarker and therapeutic target. Pharmaceuticals. 2017;10:39. 10.3390/ph10020039 PMC5490396 28379203

