# Supplementary material for: Expression and prognostic value of TRPM7 in canine mammary tumours
Source: Vet Comp Oncol. 2021 May 4;19(3):510–7. doi: 10.1111/vco.12689 (PMC8453503; doi:10.1111/vco.12689)
Supplement: Supplementary file 1 — Supplementary Table 1 The average cytoplasmic intensity scores for the selected region were calculated based on the thresholds [file VCO-19-510-s001.docx]

**Supplementary Table 1**. The average cytoplasmic intensity scores for the selected region were calculated based on the thresholds

| Parameter | Default | Description |
| --- | --- | --- |
| (1+) Threshold | 210 | Weakly positive |
| (2+) Threshold | 180 | Moderately positive |
| (3+) Threshold | 150 | Strongly positive |
